# Alternative mechanisms alter the emergent properties of self-organization in mussel beds

**DOI:** 10.1098/rspb.2012.0157

**Published:** 2012-03-14

**Authors:** Quan-Xing Liu, Ellen J. Weerman, Peter M. J. Herman, Han Olff, Johan van de Koppel

**Affiliations:** 1 Department of Spatial Ecology, Royal Netherlands Institute for Sea Research, PO Box 140, 4400 AC, Yerseke, The Netherlands; 2 Netherlands Institute of Ecology (NIOO-KNAW), Droevendaalsesteeg 10, 6708 PB, Wageningen, The Netherlands; 3 Community and Conservation Ecology, Centre for Ecological and Evolutionary Studies, University of Groningen, PO Box 11103, 9700 CC, Groningen, The Netherlands

**Keywords:** spatial self-organization, emergent property, ecosystem functioning, critical slowdown, mussel beds

## Abstract

Theoretical models predict that spatial self-organization can have important, unexpected implications by affecting the functioning of ecosystems in terms of resilience and productivity. Whether and how these emergent effects depend on specific formulations of the underlying mechanisms are questions that are often ignored. Here, we compare two alternative models of regular spatial pattern formation in mussel beds that have different mechanistic descriptions of the facilitative interactions between mussels. The first mechanism involves a reduced mussel loss rate at high density owing to mutual protection between the mussels, which is the basis of prior studies on the pattern formation in mussels. The second mechanism assumes, based on novel experimental evidence, that mussels feed more efficiently on top of mussel-generated hummocks. Model simulations point out that the second mechanism produces very similar types of spatial patterns in mussel beds. Yet the mechanisms predict a strikingly contrasting effect of these spatial patterns on ecosystem functioning, in terms of productivity and resilience. In the first model, where high mussel densities reduce mussel loss rates, patterns are predicted to strongly increase productivity and decrease the recovery time of the bed following a disturbance. When pattern formation is generated by increased feeding efficiency on hummocks, only minor emergent effects of pattern formation on ecosystem functioning are predicted. Our results provide a warning against predictions of the implications and emergent properties of spatial self-organization, when the mechanisms that underlie self-organization are incompletely understood and not based on the experimental study.

## 1. Introduction

Over the past decade, a number of studies have reported on self-organized spatial patterns from a wide range of ecosystems [1]. Examples include arid ecosystems [2], savannahs [3,4], tidal freshwater marshes [5], intertidal mudflats [6] and mussel beds [7,8]. Spatial patterns have been deemed important as changes in their shape can be used as early signals for forthcoming catastrophic shifts to alternative, degraded regimes [9,10].  Furthermore, self-organized spatial patterns are predicted to have important implications for ecosystem functioning, in terms of increased productivity and resilience [1,4,7], maintaining high biodiversity [11,12] and sediment accumulation [6,13]. Understanding how spatial patterns affect ecosystem functioning is critical for the conservation of these unique and highly valued ecosystems.

Although many studies put forward mechanistic explanations for observed patterns based on proposed interactions between organisms and environmental variables, empirical support is often limited, and alternative mechanisms are only rarely considered. However, alternative mechanisms that equally explain the observed spatial pattern are often easily derived [5,14]. The choice of mechanistic formulation, however, can potentially have important consequences for the emergent effects of patterning as predicted by the models, limiting our understanding of whether and how spatial patterns influence ecosystem functioning. Hence, it is imperative to consider and compare alternative model formulations when addressing the emergent effects of spatial patterns on the functioning of natural systems.

Here, we propose and compare two models with alternative mechanistic explanations of self-organized spatial patterns in young mussel beds on intertidal flats. Large aggregations of the blue mussel (*Mytilus edulis*), called mussel beds, are commonly found in intertidal soft-bottom substrates, and vary in size from ten to one hundred thousand square metres [15–17]. The adaptive value of aggregation mainly relates to reduction of wave disturbance and predatory losses [18–20], improving mussel survival. Aerial surveys of mussel beds in the Wadden Sea revealed that aggregation can result in the formation of regular patterns [7]. Van de Koppel *et al*. [7] explained the formation of these patterns by the interplay of local facilitation and large-scale competition for algae, inducing spatial self-organization. This model assumes that facilitation between mussels, resulting from aggregation, reduces losses owing to predation and wave dislodgement, as mussels bind to each other using byssus threads to form strong clusters and mats. We will refer to this model as the ‘decreased losses feedback’.

The blue mussel is an ecosystem engineer that substantially affects its physical surroundings [21–23]. Mussels influence both the deposition and transport of fine sediment towards the mussel bed [15,24,25], which leads to the formation of hummocks underneath patches of mussels. On top of these elevated hummocks, mussels may have improved access to algal food, as the decreased water depth increases water flow, and thereby locally alleviates algal depletion. This will lead to a higher net growth on the hummocks, and hence constitutes an alternative positive feedback mechanism. We will refer to this hypothesized process as the ‘sediment accumulation feedback’.

In this paper, we construct and compare alternative models based on either the decreased losses feedback or the enhanced sedimentation feedback. First, we present field observations indicating the presence of feedback via sediment accumulation, as an alternative to the decreased losses feedback assumed earlier. We then examine how the alternative mechanisms affect the spatial shape of the predicted patterns, and use bifurcation analysis to study how the properties of the patterned equilibrium change with decreased algal supply rates. We focus our analysis on the emergent properties of the predicted patterns in terms of the carrying capacity for mussels, vulnerability to catastrophic shifts and the resilience to disturbances. We discuss the implications of our results for the study of the emergent effects of spatial self-organization in ecological systems.

## 2. Empirical observations

### (a) Methods

We tested the hypothesis that on top of elevated hummocks, mussel biomass or density is higher as mussels have improved access to algal food, against the alternative hypothesis that biomass or density is highest at the up-flow side of each mussel patch, as they experienced a minimal level of competition from mussels upstream. For this, we conducted a field study on a striped mussel bed near Schiermonnikoog, The Netherlands (53.46798° N, 6.22494° E). We sampled mussels at the front, middle and rear position of a mussel hummock (samples covered about 0.0314 m^2^) and took the samples to the laboratory for analysis. Relative elevation was determined using a Trimble laser level (www.trimble.com). Dry biomass per square metre and individual weight of five mussels were determined after drying at 80°C for 28 h.

### (b) Results

**Figure 1.**
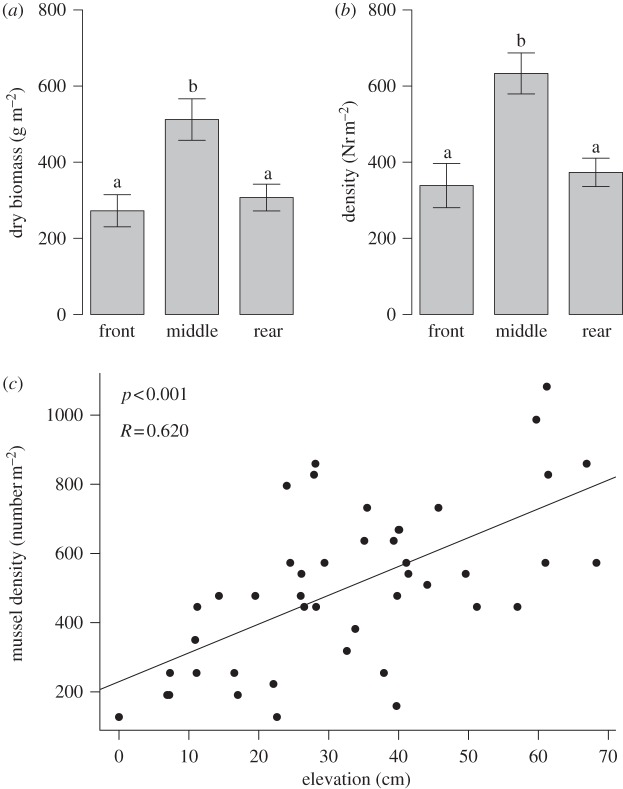
(*a*,*b*) Differences in mussel density/biomass among the front, middle and rear positions within a mussel band, where error bars denote ±1 s.e.m. The characters on top of the bars denote significant differences between the treatments based on Tukey's honest significant difference post hoc analysis of variance. (*c*) Effect of sediment accumulation on mussel density and biomass.

The results showed that mussel density and biomass are significantly higher in the middle compared with the front or rear positions (figure 1*a,b*; one-way ANOVA, biomass: *F*_2,30_ = 8.4258, *p* < 0.001; density: *F*_2,30_ = 10.153, *p* < 0.001). This suggests that mussel survival or growth is higher at the top of a hummock, compared with the sides. Moreover, regression analysis of the relation between mussel density and sediment elevation reveals that sediment elevation is positively related to mussel density (figure 1*c*; analysis with general linear model, *p* < 0.001). These results point at the possible importance of sediment accumulation and subsequent hummock formation for mussel growth. Our results suggest that sediment accumulation is a possible alternative mechanism for local facilitation, as mussels improve their own growth and that of local conspecifics by accumulating sediment underneath them.

Here, we propose a new hypothesis, based on these empirical observations, which suggests that a positive feedback exists between sediment accumulation and mussel growth. A likely mechanism for this is increased water velocity at the top of hummock compared with the bottom of the hummock, as the water is forced through a smaller cross-section. This will lead to an increase of the local algal supply rate at the top compared with the bottom of the hummock. Moreover, this can enhance water renewal and vertical transport, which is determined by flow-induced turbulence [26,27]. Our hypothesis is in close agreement with the current understanding of hydrodynamics, which predicts that the top of the hummock experiences a faster water velocity than the bottom of the hummock [26,27].

## 3. Models of pattern formation in mussel beds

**Figure 2.**
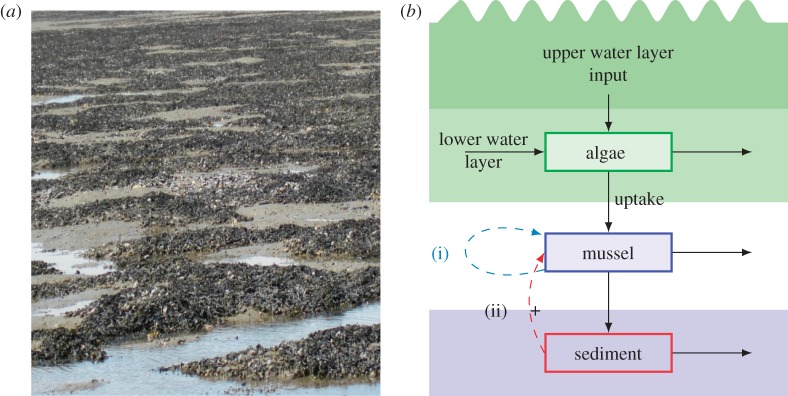
Landscape of mussel banding and two possible mechanisms. (*a*) Photograph of banded mussel patterns, clearly showing the mussels on top of hummocks of accumulated sediment. (*b*) Schematic of the state variables and the non-spatial processes in the models. The new model consists of three state variables, represented by compartments in the diagram: algae concentration in the lower water layer, mussel biomass and sediment accumulation. Algae concentration in the benthic boundary layer is determined by the exchange with the upper water layer, the tidal flow and the consumption by the mussels. Arrows indicate flows of mussel biomass, algae and sediment from one compartment to the other. (i) and (ii) represent the two alternative mechanisms: mussel aggregation as a direct promoter and sediment accumulation as an indirect promoter, respectively.

Here, we develop and compare two spatially explicit models that formalize alternative mechanisms for the local facilitation process. The first mechanism involves a positive effect of mussel density on mussel survival rates, as clumping and attachment to other mussels with byssus threads reduces chances of predation and wave dislodgement. This mechanism is the central hypothesis of a prior paper on self-organized pattern formation in mussel beds [1,7]. The second mechanism involves a positive relation between feeding efficiency and sediment accumulation, which is based on the results of the field survey presented in the prior section. Both models involve the same large-scale negative feedback arising from algal depletion by the mussels. Figure 2*b* shows a schematic of the state variables and the non-spatial processes that are considered in this study, where the two labels (i) and (ii) denote the two alternative positive feedbacks as mentioned above.

### (a) A general spatial model

We start by constructing a general continuous-time spatial model for spatial pattern formation in mussel beds. On intertidal flats, the filtration of algae by mussels occurs mostly in the lower water layer [28,29]. Algal food is supplied to this layer by influx of algae from the upper water layer and by lateral transport of algae through tidal currents. Under the simplifying assumption that the upper water layer is not affected by the consumption of the mussels [7], changes in the concentration of algae *A* in the lower water layer overlying a particular location (*X,Y*) on the mussel bed can be expressed as3.1
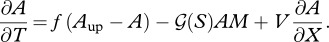
Here *A*_up_ is the concentration of algae in the surface layer; *f* is the rate of mass transfer between the benthic boundary layer and the rest of the water column. The second term represents the uptake of algae by the mussels, assuming a linear relation between mussel uptake and algal concentration, where the coefficient 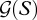 describes the specific algal consumption rate of a mussel per unit algae, as a possible function of the accumulated amount of sediment. The algae influx is driven by advection induced by tidal currents, which is represented by the gradient operator *∂A*/*∂X* multiplied by the advection constant *V*.

Mussel growth and mortality in soft-sediment mussel beds are mostly determined by the availability of algal food and by wave dislodgment and predation. We therefore describe the rate of change of mussel biomass per square metre with the expression:3.2
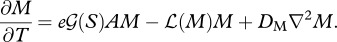
Here, the parameter *e* describes the conversion constant of ingested algae to mussel biomass. The second term is used to represent all losses in biomass owing to wave dislodgment and mortality (e.g. predation). Furthermore, the movement of mussels is described by the classical diffusion approximation, where diffusion is a linear function of the Laplacian operator ∇^2^*M* with diffusion coefficient *D*_M_.

### (b) Reduced losses model

A large number of papers in the literature show that by generating clumps, mussels can reduce their losses to predation and wave dislodgement. Following this observation, van de Koppel *et al*. [7] proposed a scale-dependent mechanism in which locally increased density of mussel reduces mussel mortality, which generated a local positive feedback. The relation between loss rate and mussel density in equation (3.2) was given by3.3
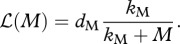


Here, *k*_M_ is the value of mussel biomass at which mortality is half-maximal, and *d*_M_ is the maximal *per capita* mussel mortality rate. The concept and algorithm details and descriptions of the coefficients can be found in [7] and electronic supplementary material, table A1 in appendix A. This model assumes a constant algal uptake coefficient (e.g. 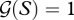).

### (c) Sediment accumulation model

Below, we construct an alternative model that includes the feedback between sediment accumulation induced by the mussels and improved uptake of algae by these mussels, reflecting the results of our field survey. First, we need to describe the rate of accumulation of sediment as a function of local sedimentation and erosion processes. We assume that sedimentation is mostly the result of the filtration activities of mussels, while erosion is proportional to the amount of sediment present:3.4
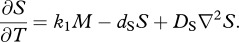
Here *S* is defined as the sediment deposited by the mussels on top of the pre-existing tidal flat surface,  *k*_1_ describes the deposition of sediment in the form of pseudofaeces per unit mussels, while *d*_S_ describes the proportional rate of erosion of sediment. Sediment is assumed to disperse in a diffusive manner, proportional to a diffusion constant *D*_S_, because of the effects of water flow and hydraulic diffusivity, where the dispersive scale of sediment is greater or equal to that of the mussels.

We now have to include in the model that sediment accumulation enhances mussel growth by increasing water flow rate, thereby enhancing algal availability. Rather than explicitly modelling water flow over a morphologically dynamic landscape, we adopt a simplifying approach where the specific uptake function 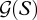 increases with sediment elevation (i.e. 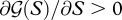) up to a maximum *c* (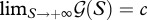, where *c* > 0). Thus, the uptake feedback is modelled as a monotonously increasing function of sediment elevation; the higher the elevation, the higher the uptake rate and the more algae are consumed by the mussels. The explicit dependence of sediment accumulation is chosen as 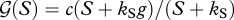, where *c* and *g* are constant. Parameter *c* defines the maximum uptake rate (per hour), *k*_S_ is the saturation constant of sediment accumulation (in centimetres per square metre) and *g* sets the proportion of *G*(*S*) that is attained when the sediment level is zero, and thus equal to the background sediment level (dimensionless). Hence, when *S* → 0; 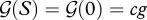. As *g* gets closer to 1, the uptake coefficient *G*(*S*) levels off to a maximum. A small *g* imposes a stronger positive-feedback effect of the sediment on the uptake rate. This scenario closely follows our assumption that the positive effect of sediment accumulation acts through the hummock development. With this mechanism, we assume a constant mussel loss rate (e.g. 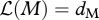). First, we construct a null model, which includes the algae, mussel and sediment, but not the positive feedback of sediment accumulation (i.e. 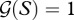). It becomes a trivial model by removing the redundant variable sediment under this specific condition. Of course, there is no pattern solution. Therefore, we do not study it further.

The mathematical formulation of the reduced loss model and the sediment feedback models are presented in table 1. Electronic supplementary material, table A1 in appendix A provides an overview of the parameter values used their units, and explanation. The estimation of parameter values is based on previous studies and is explained in electronic supplementary material, appendix A.

**Table 1.** Mathematical formulations of the decreased losses feedback (DLF) model and sediment accumulation feedback (SAF) model, as well as the number of possible states predicted by the model when not patterned.

model	equations^a^	feedback	stable^b^
DLF model			
algae	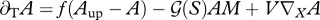	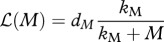	monostability
mussel	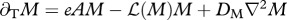	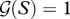	
SAF model			
algae	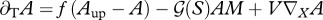	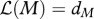	
mussel	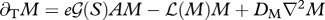	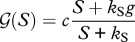	bistability
sediment	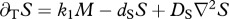		

^a^The subscript T denotes the partial time derivative.

^b^Here, the stability indicates the ordinary differential equations system rather than partial differential equations system. However, the stability of patterned solutions can be obtained by AUTO-07p and Wavetrain software [30].

### (d) Model analyses

To investigate the effect of the alternative feedback mechanisms on spatial pattern formation, we performed a two-dimensional numerical simulation of the models derived above. The spatial patterns result from Euler integration of the finite-difference equations with discretization of the diffusion and advection operators [31]. The models' predictions were examined for different grid sizes and physical length. We used a rectangular spatial grid with a unidirectional water flow in the *x* direction driving the advection. We adopted periodic boundary conditions in the flow direction to mimic predepletion of the water by the bed surrounding the simulated domain. Reflecting boundaries were adopted in the other direction. Starting conditions consisted of a homogeneous equilibrium with a slight random perturbation.

An important focus is the question of whether the models' predictions differ with regard to the effects that patterns have on the functioning of the mussel bed, in terms of (i) bed-wide production of biomass and (ii) resilience to disturbances. To answer the first question, we studied the difference in predicted mussel biomass at equilibrium, comparing the average biomass in the patterned and the homogeneous state. This difference, however, is sensitive to changes in, for instance, the algal input concentration (*A*_up_), which is the parameter that clearly distinguishes different intertidal habitats. We therefore analysed the changes in the models' predictions with respect to this parameter using spatial bifurcation analysis [32,33], which is an effective method to study the emergence of spatial patterns and their implications in relation to forcing parameters [33–35]. Bifurcation analyses were performed using the bifurcation package AUTO-07p [36].

The second question concerns the effects that the alternative mechanism has on resilience, in terms of the time required to return to equilibrium. A recent study [7] has shown that in mussel beds, self-organized spatial patterns improve the resilience to perturbation. To study the effect of the alternate mechanisms on ecological resilience, we compared the recovery time after perturbation with the pre-perturbation equilibrium in three simulation cases, following the previous study of van de Koppel *et al*. [7]. Here, the recovery time (*τ*) is defined as the period taken for mussel biomass to reach 0.97 *M*_eq_ from a 0.9 *M*_eq_ (or 0.5 *M*_eq_) perturbation from the previous equilibrium, where *M*_eq_ refers to equilibrium biomass. We implement the simulation based on the spatial scale of two wavelengths, and adopt bidirectional advection for the algal differential equation, where advection direction switches sign every 6 h. Bidirectional advection includes both the ebb tide and flood tide processes, which leads to the development of stationary patterns that allow for measurement of the return time to equilibrium. Both the unidirectional and bidirectional set-up generate qualitatively similar results, but equilibrium biomass in the unidirectional set-up is intrinsically fluctuating because of the movement of the banded solution.

## 4. Results

### (a) Results of model analysis

**Figure 3.**
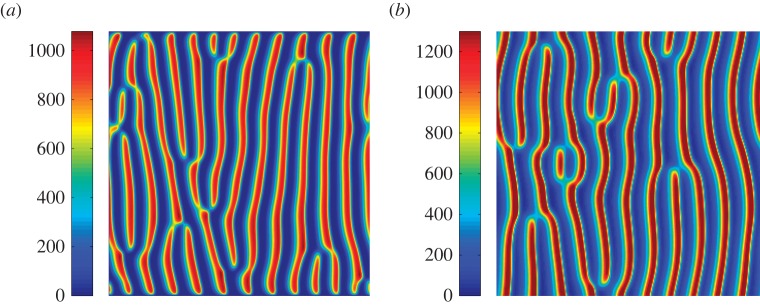
Two-dimensional numerical model simulations mimicking mussel banding, representing an aerial view of the mussel bed on a square intertidal flat area of 50 × 50 m. For the simulation, the parameter values are reported in the electronic supplementary material, appendix A. (*a*) The decreased losses feedback model that uses mussel aggregation as promoter. (*b*) The sediment accumulation feedback model, where sediment accumulation is used as promoter.

Comparison of the predictions of the models reveals that very similar spatial patterns develop, irrespective of the type of facilitative interactions between mussels (figure 3). In both models, a regular pattern develops of mussels ordered in bands, oriented perpendicular to the orientation of the tidal flow. The new sediment accumulation model reveals a similar pattern of sediment elevation, the additional component relative to the reduced-losses model, reflecting the pattern within the mussels. This results in a landscape of regularly placed sediment hummocks with high mussel biomass on top of the hummocks (figure 3), in which the ridges and hollows are again oriented perpendicular to the direction of the tidal flow. In all models, the formation of patterns depends strongly on the presence of initial variation in biomass, sediment level or algal concentration, as no patterning develops if the starting conditions are entirely homogeneous. This reveals that spatial interactions are a key mechanism explaining pattern formation. Hence, the patterns that are generated by the model indicate that growth facilitation via accumulation of sediment is a valid alternative explanation for the observed patterns in mussel beds on intertidal flats.

**Figure 4.**
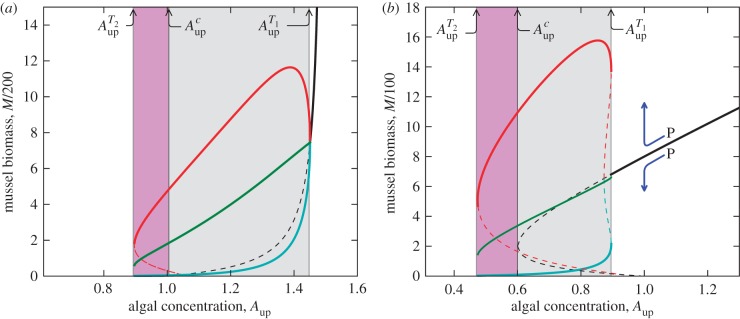
Bifurcation diagram of mussel biomass based on spatial solutions. The solutions are plotted in terms of mussel biomass *M* versus the bifurcation variable *A*_up_, which determines algal supply. All the solutions are obtained by integration of dimensionless parameters via AUTO-07p within a periodic domain, and their stability was determined using a numerical eigenvalue method, as shown in electronic supplementary material, figure S1. Solid lines mark stable portions of the branch; the coloured rectangular regions (including both the pink and the grey area) represent the spatially patterned state, characterized by striped patterns. Black lines represent the homogeneous equilibrium, red lines and cyan lines represent maximum and minimum amplitude of mussel biomass in the patterned equilibrium, respectively, and the green line represents average mussel biomass within the simulated domain. (*a*) Decreased losses feedback model and (*b*) sediment accumulation feedback model.

Bifurcation analysis revealed a clear effect of algal supply on the characteristics of the patterned mussel beds, where the presence of patterning strongly depended on the value of algal input *A*_up._ Both the decreased losses model and the sediment accumulation model have a homogeneous, non-zero state for all *A*_up_ values above a minimum 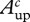 (see electronic supplementary material, appendix A for mathematical solutions of the homogeneous states). This state is represented by a solid black line in figure 4. Mussel biomass and sediment accumulation decrease monotonously with declining algal supply rate until mussels cannot maintain themselves on the intertidal flat for lack of food. For both models, this uniform state is stable (solid line) at algal supply values greater than a critical value 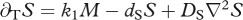. Below this threshold value, the uniform state becomes unstable to spatially heterogeneous perturbations, referred to as Turing instability in the literature [37]. This implies that small spatially heterogeneous disturbances are inflated, and regular spatial patterns will emerge, as shown in figure 3. In both the decreased losses feedback and sediment accumulation feedback models, the patterned system is globally stable up to algal supply rates at 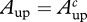. When algal concentration is in the range 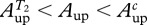, the system has two attracting states, where one state is characterized by spatial patterns (solid red line in figure 4; represents maximal mussel biomass), while the other is a uniform state with no mussels. This result suggests that spatial self-organization allows mussels to persist at algal concentrations that would not permit survival of mussels in a homogeneous bed. Beyond the last threshold, the patterned state collapses and only a homogeneous state without mussels is found (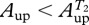). Here, the thresholds 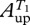 and 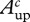 can be derived analytically following a standardized linear analysis [37]. However, the critical value 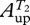 is impossible to predict with the standardized linear analysis of pattern solutions because it is caused by a spatially nonlinear effect.

The bifurcation analyses reveal a striking difference between the decreased losses model and the sediment accumulation model in terms of the implications of spatial self-organization for ecosystem functioning. In the decreased losses model, average mussel biomass is much higher in the patterned equilibrium compared with the homogeneous equilibrium (cf. figure 4*a*, green lines versus black lines) for all parameter values where stable patterns are predicted. In the sediment accumulation model, this difference is much smaller, or even reversed, depending on the algal input concentration. This points at an important difference between the decreased losses model and sediment accumulation model, in that increased production, which is considered an important emergent effect of spatial self-organization on ecosystem functioning in the decreased losses model [7], is virtually absent in the sediment accumulation model.

### (b) Ecological resilience

**Figure 5.**
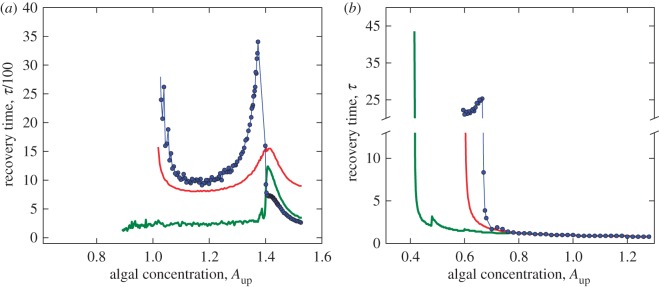
Results of post-perturbation recovery for the two alternative mechanisms, using the models listed in table 1. The recovery time (*τ*) of a mussel bed is determined following a perturbation in which the density of mussels is reduced by 10%. The green solid lines represent a simulation where the patterns are left intact. The blue lines with circles represent a simulation where following the perturbation, the mussel beds, sediment and algae were evenly redistributed, after which a random deviation (<1%) was imposed on the biomass within each cell (near homogeneous). The red lines represent a simulation with which the mussels, sediment and algae were homogenized in space, leaving no spatial variability (homogeneous). The parameters are as in electronic supplementary material, table A1, apart from algal concentration *A*_up_. (*a*) Decreased losses feedback model and (*b*) sediment accumulation feedback model. Note that the noise observed in the graphs is caused by discrete nature of both space and time, while the jump in the patterned solution in (*b*) is caused by a shift in frequency in the solution. Green line, pattern intact; red line, homogeneous; circles with continuous line, near homogeneous.

Comparison of the models shows a remarkable difference between the reduced losses model and both sediment accumulation models with respect to the ecological resilience to a 10 and 50 per cent reduction in biomass (see electronic supplementary material). We compared simulation trajectories, where the patterns were left intact with simulations where mussel biomass was nearly homogenized, and studied the time the system needed to return to the patterned equilibrium. The recovery time of these two simulations we in turn compared with the recovery time in the homogeneous system. First of all, in all models, mussel biomass recovered much faster after a perturbation if the patterns were left intact than if the biomass was redistributed near-homogeneously. This difference increases as the critical threshold 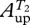 is approached (figure 5*a*). Qualitatively similar differences between simulations were found in the model with the sediment accumulation feedback model (figure 5*b*).

We found a remarkable quantitative difference in the recovery time between the reduced losses model and the sediment accumulation model. With both models, recovery times were found to increase in the homogeneous state when *A*_up_ approached the critical thresholds 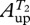 and 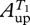, a phenomenon referred to in the literature as critical slowing down [10]. Critical slowing down describes the phenomenon that recovery rates from small perturbations tend to zero when a tipping point is approached [38,39]. Strikingly, this phenomenon was not observed in the patterned state of the reduced losses model, when, after a perturbation, the patterns were left intact (figure 5, green line). This implies that critical slowing down, a process that severely impairs resilience, was buffered by the aggregation feedback that is central to this model. Remarkably, this phenomenon was not present in the sediment accumulation model, as the recovery time was found to increase as 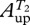 was approached. This difference highlights that possible use of critical slowing down as an indicator of proximity of catastrophic shifts depends strongly on the mechanisms that underlie spatial pattern formation.

### (c) Robustness of the analysis

Our model still includes a very simplified description of the effect of enhanced sediment accumulation on the uptake and growth of the mussels. Specifically, we relate uptake to absolute sediment accumulation, while relative sediment elevation of the mounts with respect to the surrounding sediment could be an alternative explanatory variable, especially when the entire sediment bed increases in elevation. For this reason, we have also analysed a second model in which growth stimulation is not a function of the absolute sediment accumulation *S*, but of the accumulation relative to the average surroundings, using a feedback function 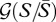 instead of the 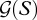 in the sediment model. Here, 
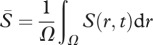
 denotes the average accumulation of sediment over the entire mussel habitat *Ω*. Although different in detail, this model again predicts similar patterns, but very different effects of the patterns on ecosystem functioning relative to the reduced losses model (see electronic supplementary material, figure S2 in appendix A). Hence, despite the simplicity of the models we used, they clearly point to the sensitivity of the predicted emergent properties to the underlying mechanism.

## 5. Discussion

The process of spatial self-organization is the central explanation for the occurrence of regular or otherwise coherent spatial patterns in ecosystems lacking underlying abiotic heterogeneity [1]. However, most theoretical studies currently focus on the explanations for observed spatial patterns, following the first mathematical model proposed by Klausmeier [2]. Typically, empirical verification of the hypothesized mechanisms in real ecosystems is rare, and only few studies consider alternative explanations for observed patterns in ecology [14]. In this study, we compare alternative mechanisms driving the formation of self-organized regular patterns in mussel beds. Mussel bed patterning is hypothesized to result from long-range inhibition by depletion of algae, and local facilitation between mussels [7]. The first mechanism, previously proposed in the literature, assumes that local facilitation results from mutual protection among mussels against predation and wave dislodgement as mussels connect by means of byssal threads, directly lowering mussel loss [7,20,40]. Based on new empirical evidence, we put forward a second, alternative facilitation mechanism that assumes that mussels promote their growth rate by stimulating the formation of sediment hummocks on which feeding is more efficient. Key in this process is the stimulation of sediment accumulation via excretion of pseudofaeces [15]. Here, we compare both mechanisms as alternative explanations for self-organized pattern formation in mussel beds.

Our results show that very similar spatial patterning emerges under both mechanisms. Hence, observation of the characteristics of the spatial pattern will not disqualify either of the proposed mechanisms. However, the models predict a strikingly different effect of spatial self-organization on ecosystem functioning, especially in terms of established mussel biomass in equilibrium. The bifurcation analysis reveals that in the decreased losses model, spatial self-organization causes a large increase of mussel biomass relative to that predicted for homogeneous beds, over an extensive range of parameter values. This prediction can be explained by the local facilitation process within mussels, in which the mussels attach themselves to each other with byssal threads, thereby reducing predation or wave dislodgement. Conversely, the analysis of the sediment accumulation model reveals only a minor or even negative effect of pattern formation on bed-wide production, especially when algal concentration in the upper layer is high. Only when algal concentration is insufficient to support mussels in a homogeneous bed, but is high enough to support a patterned bed (within the range 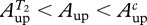; figure 4), is there a significant effect of patterning on bed-level production. Hence, we found a clear and strong effect of the mechanism that underlies pattern formation on the emergent properties of these patterns for the mussel bed ecosystem.

A similar difference was found when the implications of pattern formation for the resilience to disturbances were analysed. In the reduced losses model, patterning strongly reduced the time needed for the system to return to equilibrium following a disturbance that imposed a 10 per cent reduction of mussel biomass. Moreover, while the homogeneous system showed a significant reduction of resilience at the edges of the parameter range, where patterns were found (a phenomenon that is called critical slowing down), no such reduction was observed in the patterned state; spatial patterning seemed to nullify the phenomenon of critical slowing down. This effect, however, was completely absent in the sediment accumulation model. Hence, the effects of patterning on resilience seem to depend strongly on the mechanisms that underlie the patterning, similar to what is predicted for bed-wide production.

A very conspicuous feature of patterned mussel beds is the co-occurring patterning in the geomorphic landscape, caused by the excretion of mud particles by the mussels, which generate mounds that are elevated on average 30 cm above the ambient sediment. The results of our field experiment clearly point out that on these mounts, a higher density of mussel biomass persists. We hypothesize that the higher density is most probably explained by improved growth conditions, caused by increased water flow rate over raised mounts. Alternative explanations such as increased recruitment on top of the hummocks or active or passive movement can probably be excluded. First, most mussel beds develop from a near-even spread of larvae on unpatterned sediment. Second, passive movement would probably remove the mussels from the mounts as mounts catch more wave action. Finally, active movement could contribute to the accumulation of mussels on the hummocks, but prior observations [8] revealed that mussels rarely move actively for more than 10 cm, excluding this as a reasonable mechanism for aggregation on the top of the hummocks. Our hypothesis is supported by a recent study on the effects of elevation on oyster growth, where both natural and artificial oyster reefs with high elevation produced more spat and contained more adults than reefs with low elevation [41]. Despite this, there is also a significant body of evidence that reveals that increased density reduces mussel losses, in support of the decreased losses mechanism. This mechanism, however, acts at the scale of the 5–10 cm clumps that mussels generate by aggregating, and is a key mechanism in the formation of the small-scale labyrinth-type patterns that mussels generate within the larger-scale banded patterns [8,42]. Hence, for the larger-scale banded patterns with 5–10 m wavelength that are found in mussel beds at many intertidal flats, local positive feedback between increased accumulation of sediment and improved growth conditions for mussels is the most likely mechanism.

Critical slowing down (e.g. a longer return time to equilibrium following disturbance) is one of the most prominent early-warning indicators for the proximity of catastrophic tipping point in ecological systems [10]. A recent study revealed that in arid systems with regular spatial patterns, critical slowing down is a prelude to a tipping point when forcing variables change [43,44]. This prediction is confirmed in the sediment accumulation model, which reveals an increase in the return time in the patterned state when a tipping point is approached, similar to what is found for the homogeneous state. The decreased losses model, however, shows a complete absence of critical slowing down, as the return time to equilibrium remains constant for nearly the entire parameter range where spatial patterns are predicted. This emphasizes that the phenomenon of critical slowing down is also dependent on the specific mechanism that is involved, in turn warning against a rapid acceptance of critical slowing down as a leading indicator for the proximity of tipping points when the empirical support for the mechanisms behind spatial patterns is weak.

In the past decade, large bodies of theoretical studies have appeared in the literature explaining spatial patterns observed in a wide variety of ecosystems. Often, these studies were based on a single explanatory mechanism, based on the intuition of the modeller rather than on a firm empirical basis, and assuming that process could be derived from pattern. Our study emphasizes that for mussels, alternative mechanisms can equally well explain the same spatial pattern, and that models, at most, give insight into possible mechanisms, rather than accurate understanding of mechanisms of pattern formation. Moreover, alternative model formulations can provide contrasting predictions of the emergent properties of spatial patterning for ecosystem functioning, and of the response of ecosystems to changing conditions. Hence, for a full understanding of the importance of spatial patterns in driving ecosystem characteristic and dynamics, alternative mechanisms should be investigated, and their assumptions validated in field studies.

The distinction between observation of patterns and inference of their underlying mechanisms, as put forward in this paper, has implications that go beyond the system studied here. For instance, since chaos was discovered by May in the 1970s from a simple population model [45,46], mathematical models have shown that complex chaotic dynamics can be generated by several different mechanisms, including competition for limiting resources [47,48], predator–prey interactions [49] and food-web dynamics [50]. Another obvious example is the mechanisms that underlie power laws in ecological systems. There are many different processes, including interactions between large-scale resource constraints and small-scale facilitation [51], grazing disturbance [52] and environmental disturbance [53,54], that can generate similar power law patterns. Our study highlights that an experimental approach to infer the mechanisms that underlie observed patterns might require us to revisit theoretical underpinnings that we have often taken for granted.
